# 1‐Deoxynojirimycin attenuates pathological markers of Alzheimer's disease in the in vitro model of neuronal insulin resistance

**DOI:** 10.1096/fj.202302600R

**Published:** 2024-07-09

**Authors:** Isabella Supardi Parida, Soo Takasu, Junya Ito, Takahiro Eitsuka, Kiyotaka Nakagawa

**Affiliations:** ^1^ Laboratory of Food Function Analysis, Graduate School of Agricultural Science Tohoku University Sendai Japan; ^2^ Schizophrenia Research Project, Department of Psychiatry and Behavioral Sciences Tokyo Metropolitan Institute of Medical Science Tokyo Japan; ^3^ Laboratory of Pharmaceutical Analytical Chemistry Gifu Pharmaceutical University Gifu Japan

**Keywords:** 1‐deoxynojirimycin, Alzheimer's disease, amyloid, insulin‐degrading enzyme, neuronal insulin signaling, tau

## Abstract

Insulin resistance, the hallmark of type 2 diabetes mellitus (T2DM), has emerged as a pathological feature in Alzheimer's disease (AD). Given the shared role of insulin resistance in T2DM and AD, repurposing peripheral insulin sensitizers is a promising strategy to preserve neuronal insulin sensitivity and prevent AD. 1‐Deoxynojirimycin (DNJ), a bioactive iminosugar, exhibited insulin‐sensitizing effects in metabolic tissues and was detected in brain tissue post‐oral intake. However, its impact on brain and neuronal insulin signaling has not been described. Here, we investigated the effect of DNJ treatment on insulin signaling and AD markers in insulin‐resistant human SK‐N‐SH neuroblastoma, a cellular model of neuronal insulin resistance. Our findings show that DNJ increased the expression of insulin signaling genes and the phosphorylation status of key molecules implicated in insulin resistance (Y1146‐pIRβ, S473‐pAKT, S9‐GSK3B) while also elevating the expression of glucose transporters *Glut3* and *Glut4*, resulting in higher glucose uptake upon insulin stimuli. DNJ appeared to mitigate the insulin resistance‐driven increase in phosphorylated tau and Aβ_1‐42_ levels by promoting insulin‐induced phosphorylation of GSK3B (a major tau kinase) and enhancing mRNA expression of the insulin‐degrading enzyme (IDE) pivotal for insulin and Aβ clearance. Overall, our study unveils probable mechanisms underlying the potential benefits of DNJ for AD, wherein DNJ attenuates tau and amyloid pathologies by reversing neuronal insulin resistance. This provides a scientific basis for expanding the use of DNJ‐containing products for neuroprotective purposes and prompts further research into compounds with similar mechanisms of action.

## Graphical abstract

1‐Deoxynojirimycin (DNJ) promotes phosphorylation of AKT and downstream GSK3B (a major tau kinase) in insulin‐resistant SK‐N‐SH, resulting in lower tau phosphorylation. Simultaneously, DNJ increases mRNA expression of IDE involved in Aβ degradation and reduces Aβ_1‐42_ accumulation.

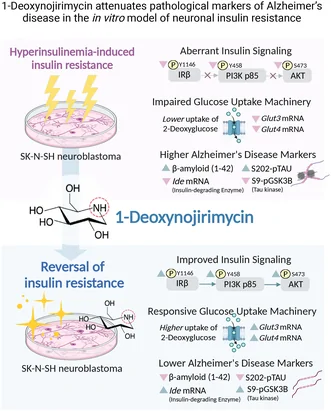

**Abbreviations**
2‐DG: 2‐deoxyglucose
AKT: protein kinase B
APP: amyloid precursor protein
DNJ: 1‐deoxynojirimycin
GLUT: glucose transporter
GSK3B: glycogen synthase kinase‐3 beta
HPLC‐MS/MS: high‐performance liquid chromatography–tandem mass spectrometry
IDE: insulin‐degrading enzyme
IRS: insulin receptor substrate
IRβ: insulin receptor
PI3K: phosphoinositide 3‐kinases
SIRT1: Sirtuin 1

## 1 INTRODUCTION

Insulin resistance, the main feature of type 2 diabetes mellitus (T2DM), is characterized by the inept response of tissues to insulin stimulation. While mainly associated with metabolic tissues, recent findings have underscored the pivotal role of brain insulin signaling for neuronal function.^1^ Consequently, brain insulin resistance emerges as a risk factor for neurodegeneration, as is the case for Alzheimer's disease (AD). The post‐mortem analyses of AD patients revealed shifts in mRNA transcripts, protein expressions, and activation of the insulin/PI3K/AKT signaling^2^, ^3^, ^4^ were associated with the cognitive decline and typical increase in tau and amyloid deposition seen in AD.^5^, ^6^

Among the proposed mechanisms linking brain insulin resistance and AD, two scenarios stand out. The first suggests that impaired insulin/IR/PI3K/AKT signaling pathway increases the catalytic activity of GSK3B, a tau kinase, resulting in tau hyperphosphorylation and formation of neurofibrillary tangles (NFTs).^7^, ^8^ The second scenario argues that impaired insulin signaling diminishes the capacity of the insulin‐degrading enzyme (IDE) to degrade β‐amyloid (Aβ) and accelerates senile plaque formation.^9^, ^10^ Both NFTs and senile plaques are hallmark features of AD, and they exacerbate the existing neuronal insulin resistance, thus creating a positive feedback loop that expedites cognitive decline.^11^ Given that insulin resistance is a shared factor in T2DM and AD, repurposing peripheral insulin sensitizers is a viable approach to preserve brain insulin sensitivity and delay the onset of AD.

1‐Deoxynojirimycin (DNJ), an iminosugar present in mulberry (*Morus alba* L.) leaf^12^ and growth media of some *Bacillus* strains,^13^, ^14^ is a fitting candidate for the above purpose. Extensive studies have reported the pharmacological effects of DNJ for non‐communicable metabolic diseases and identified probable mechanisms of action.^15^ Among them, the insulin‐sensitizing effect of DNJ on metabolic tissues has been consistently linked to better cardiometabolic parameters and reversal of diabetes in murine models of diabetes and prediabetes.^16^, ^17^ Notably, DNJ increased the expression of insulin signaling genes *Irs1*, *Akt2*, and *Pten* in white adipose tissue of diet‐induced obese mice^18^ and promoted the activation of PI3K/AKT signaling and membrane GLUT4 translocation in the skeletal muscle and liver of db/db mice.^19^, ^20^ These shifts led to improved systemic insulin sensitivity, postprandial glucose regulation, and lipid processing in the above studies. Additionally, DNJ was detected in brain tissues post‐oral intake, with approximately 1.5 nmol DNJ/g tissue detected after a single oral intake of 40 μmol DNJ/kg BW,^21^ and 17.0 ± 2.5 ng DNJ/g tissue detected after repeated intake of 10 mg DNJ/kg BW for five weeks.^22^ This, coupled with its commendable safety profile,^12^, ^23^ makes DNJ a safe and viable option for short‐term and long‐term neuroprotective purposes.

Furthermore, a handful of studies have indicated the neuroprotective capacity of DNJ. For instance, studies on senescence‐accelerated mouse‐prone 8 (SAMP8) have shown the moderate potency of DNJ in reversing the age‐related decline in sensorimotor and cognitive function when given at higher doses,^24^, ^25^ though these changes were primarily attributed to enhanced BDNF/TrkB signaling and reduced hippocampal inflammation. Recent studies have also explored the benefits of DNJ derivatives for AD, with one study reported the anti‐amyloidogenic effect of N‐butyldeoxynojirimycin (miglustat) in a cell model of human AD,^26^ while others synthesized N‐alkylated DNJ derivatives^27^ and DNJ‐benzotriazole hybrids^28^ with strong inhibition towards butyrylcholinesterase, an enzyme whose increased catalytic activity during AD promotes aberrant hydrolysis of the neurotransmitter acetylcholine. These data further strengthen our hypothesis on the neuroprotective potential of DNJ.

It is important to note that the above studies were aimed at early or late‐stage AD when the loss of neuronal and cognitive function has manifested. Since some pathological processes of AD occur years before the symptoms arise—as is the case for neuronal insulin resistance—we believe that it is vital to assess the benefit of DNJ at this stage. So far, little is known about DNJ's role in neuronal insulin signaling. Hence, we used insulin‐resistant human SK‐N‐SH neuroblastoma, a representative cell model of neuronal insulin resistance, to determine the potential benefits of DNJ on impaired insulin signaling and the associated rise in AD markers. The results from this study shed new light on the neuroprotective mechanism of DNJ and provide a solid basis for its use as a preventive measure to delay the onset of AD.

## 2 MATERIALS AND METHODS

### 2.1 Materials

DNJ for cell culture experiments was isolated and purified from the cell culture medium of *Bacillus amyloliquefa*ciens DSM7 in accordance with our previous study.^14^ All‐trans‐retinoic acid (ATRA) for cell differentiation was purchased from FUJIFILM Wako Pure Chemical Corporation (Osaka, Japan). The DNJ standard for quantification using HILIC‐MS/MS was purchased from Sigma‐Aldrich Japan (Tokyo, Japan). All other materials used in this study were of analytical grade.

### 2.2 Cells and maintenance

The human neuroblastoma cell line SK‐N‐SH was obtained from RIKEN BRC (Tsukuba, Japan) and maintained in Minimum Essential Medium Eagle with alpha modification (Sigma‐Aldrich, Missouri, USA) containing 10% fetal bovine serum (FBS; Gibco™, Thermo Fisher Scientific, Massachusetts, USA) and antibiotics (100 U/mL penicillin and 100 μg/mL streptomycin; Gibco™, Thermo Fisher Scientific, Massachusetts, USA) in a humidified atmosphere containing 5% CO_2_ at 37°C.

### 2.3 Cell differentiation, induction of insulin resistance, and treatment with DNJ

SK‐N‐SH cells at 40–50% confluence were differentiated in Minimum Essential Medium‐α (MEM‐α; Gibco™, Thermo Fisher Scientific, Massachusetts, USA) containing 10 μM ATRA, with the FBS level gradually reduced from 2% to 0.5% during differentiation period. The differentiation media was replaced every 2–3 days for 14 days or until cells showed neurite outgrowth (cells with neurites longer than one cell body length). Differentiation state was confirmed by mRNA expression of three differentiation markers: *NeuroD1* (*NeuD*), *Synapthophysin* (*Syp*), and *Map2* (Figure S1). Differentiated cells were maintained in 0.5% FBS/MEM‐α for 4–5 days before induction of insulin resistance.

Insulin‐resistant (INS‐R) states were induced by treating differentiated SK‐N‐SH cells with 250 nM insulin in 0.5% FBS/MEM‐α media for 24 h. Following this, INS‐R cells were incubated in 0.5% FBS/MEM‐α containing either 2.5, 5 or 10 μM DNJ (INS‐R + DNJ 2.5/5/10) or without DNJ (INS‐R) for another 24 h. Insulin‐sensitive cells were incubated in 0.5% FBS/MEM‐α without insulin for the first 24 h, then incubated with 5 μM DNJ (DNJ 5) or without DNJ (Control) for another 24 h. The insulin and DNJ levels used in this study did not affect cell viability (Figure S2A,B).

### 2.4 Analysis of mRNA expression using real‐time quantitative reverse transcription polymerase chain reaction (RT‐qPCR)

The cells were seeded in a 6 cm dish (1 × 10^6^ cells/dish) and underwent treatment described in Section 2.3. Before RNA extraction, cells were serum‐starved for 2 h, then incubated in serum‐free MEM‐α containing 100 nM insulin for 30 min to stimulate insulin signaling. RNA was isolated using NucleoSpin® RNA kit (Macherey‐Nagel, Pennsylvania, USA) according to the kit's protocols. To form complementary DNA (cDNA) using reverse transcription, a 10 μL mixture containing 500 ng of RNA, 2 μL of PrimeScript™ RT Master Mix (Perfect Real Time) (Takara Bio Inc., Shiga, Japan), and RNase‐free H_2_O was incubated at 37°C for 15 min, heated at 95°C for 5 min, and stored at −20°C until use.

The RT‐qPCR reaction was conducted in a mixture containing 8 μL of cDNA (= 100 ng DNA template), 2 μL of forward primer (0.4 μM final concentration), 2 μL of reverse primer (0.4 μM final concentration), and 10 μL of TB Green™ Premix Ex Taq™ II (Til RNaseH Plus) (Takara Bio Inc., Shiga, Japan). Amplification and detection were performed using a Bio‐Rad CFX96 real‐time PCR detection system (Bio‐Rad Laboratories, California, USA) with the following conditions: 95°C for 10 s, 40 cycles at 95°C for 5 s, and 60°C for 30 s. The relative expressions of target genes were determined using the 2^−ΔΔCT^ method, with GAPDH as the reference. Primer sequences are listed in Table S1.

### 2.5 Western blotting (WB)

The cells were seeded in a 10 cm dish (2.5 × 10^6^ cells/dish) and underwent the treatments described in Section 2.3. Prior to the assay, cells were serum‐starved for 2 h and then incubated in serum‐free MEM‐α with 100 nM insulin (insulin‐stimulated) or no insulin (basal) at 37°C for 30 min. Cells were then scraped and sonicated in ice‐cold lysis buffer containing protease/phosphatase inhibitors (Cell Signaling Technology, Massachusetts, USA) and spun (15 000 × g, 20 min, 4°C). Protein content in supernatant was measured using Bio‐Rad protein assay dye reagent (Bio‐Rad, California, United States). After equilibrating the protein content, the extracts were mixed with 6x Sample Buffer (Tokyo Chemical Industry, Tokyo, Japan) containing β‐mercaptoethanol, boiled at 95°C for 5 min, and stored at −20°C until use.

Cellular proteins were separated by sodium dodecyl sulfate‐polyacrylamide gel electrophoresis and transferred to a polyvinylidene fluoride (PVDF) membrane (Immobilon‐P; Millipore, Massachusetts, USA) using the Trans‐Blot® Turbo™ system (Bio‐Rad, California, United States). The membranes were blocked with PVDF Blocking Reagent for Can Get Signal® (TOYOBO Co., Ltd., Osaka, Japan) for 1 h at room temperature, followed by overnight incubation with primary antibodies at 4°C and a 1 h incubation with secondary antibodies at room temperature. The list of antibodies is provided in Table S2. Protein bands were visualized by Immobilon Western Chemiluminescent HRP Substrate (Millipore, Massachusetts, USA) and captured with LuminoGraph 1 (ATTO Corp, Tokyo, Japan). Densitometry analyses were done using ImageJ.

### 2.6 Glucose uptake assay

Glucose uptake was determined based on the cellular level of 2‐deoxyglucose (2‐DG) using the Abcam Glucose Uptake Assay Kit (Colorimetric) (Abcam, Cambridge, UK). The cells were seeded in a 96‐well plate (5 × 10^4^ cells/well) and underwent treatments described in Section 2.3. Before the assay, cells were glucose‐deprived in Krebs‐Ringer‐Phosphate‐Hepes buffer containing 2% bovine serum albumin (BSA) for 40 min at 37°C and incubated for another 30 min in serum and glucose‐free Dulbecco's Modified Eagle Medium (DMEM; Gibco™, Thermo Fisher Scientific, Massachusetts, USA) containing 100 nM insulin (insulin‐stimulated) or no insulin (basal). Ten microliters of 10 μM 2‐DG were added to each well, and the cells were incubated at 37°C for another 30 min. Cells were washed with PBS to remove excess 2‐DG and lysed using Extraction Buffer (80 μL), followed by freeze‐thawing and heating (37°C, 40 min). Cell lysates were cooled on ice for 5 min, neutralized with Neutralization Buffer (10 μL), and spun (500 rpm, 1–2 min). The 2‐DG level in the supernatant was measured following the kit's protocols.

### 2.7 Measurement of β‐amyloid (Aβ) level in the cell‐conditioned medium

Cells were seeded in a 6‐well plate (3 × 10^5^ cells/well) and underwent treatment as described in Section 2.3. Three milliliters of cell‐conditioned medium were collected and mixed with 0.2% BSA and 0.075% 3‐[(3‐cholamidopropyl)dimethylammonio]‐1‐propane sulfonate to prevent Aβ adhesion to the tubes, then centrifuged at 3000 × g for 10 min at 4°C to remove debris. Aβ level was determined using Human β‐Amyloid (1–42) ELISA Kit Wako, High‐Sensitive (FUJIFILM Wako Pure Chemical Corporation, Osaka, Japan) according to the manufacturer's protocols.

### 2.8 Time‐dependent cellular uptake of DNJ

Differentiated SK‐N‐SH cells in 10 cm dishes were incubated in 0.5% FBS/MEM‐α medium containing 5 μM DNJ (37°C, 5% CO_2_). The medium was decanted at each time point (0, 6, 12, 18, 24 h), and cells were washed with cold PBS and scraped in 500 μL of water. Five hundred microliters of acetonitrile were added to the cell suspension, followed by centrifugation (10 000 × g, 10 min, 4°C). The supernatant was loaded into a strong cation‐exchange solid‐phase extraction column (strata SCX, 100 mg, Shimadzu, Kyoto, Japan) previously conditioned with 1 mL of methanol containing 0.1% formic acid. The column was washed with 1 mL of methanol containing 0.1% formic acid, and DNJ was eluted twice with 1 mL of 1 M methanolic NH_3_ solution. The eluate was spin‐dried under vacuum conditions (1500 rpm, 42°C), and the resulting solids were redissolved in 70% acetonitrile solution (200 μL) and stored at −80°C until use.

For DNJ quantification, a 10 μL aliquot of extract was subjected to HILIC‐MS/MS consisting of an ExionLC HPLC/UHPLC system and a 4000 QTRAP tandem mass spectrometer (SCIEX; Tokyo, Japan). Chromatographic separations were performed on a HILIC column (TSKgel Amide‐80, 5 μm, 2.0 mm × 100 mm; Tosoh, Tokyo, Japan) at 40°C with a flow rate of 0.2 mL/min. The gradient program was as follows: 0–2 min, 20–60% B; 2–5.5 min, 60% B; 5.5–5.6 min, 60–20% B; 5.6–10.0 min, 20%, where mobile phase A was 0.1% formic acid in acetonitrile, and mobile phase B consisted of 0.1% formic acid in water. DNJ was detected using MS/MS under positive ion modes with multiple reaction monitoring (MRM) for the transition of parent ion to product ion according to our previous method.^29^

### 2.9 Statistical analysis

Data are shown as mean ± SD, with differences among groups assessed using the analysis of variance (ANOVA) with Tukey posthoc test. Statistical analyses were performed with GraphPad Prism version 9.4.1 (GraphPad Software LLC., Massachusetts, USA), with *p* < .05 considered statistically significant.

## 3 RESULTS

### 3.1 DNJ altered gene and protein expressions related to insulin signaling in insulin‐resistant SK‐N‐SH

Like cultured adipocytes and muscle, differentiated neuroblastoma shows signs of insulin resistance when exposed to prolonged hyperinsulinemia,^30^, ^31^ making them a fitting model for studying neuronal insulin resistance. Our data show a proportionate change in the expression of genes related to insulin signaling and glucose uptake in differentiated SK‐N‐SH within 24 h of exposure to 100 nM and 250 nM insulin (Figure 1). As the latter evoked a more pronounced change, we applied this condition in the following experiments. Compared to the insulin‐sensitive cells (Control), insulin‐resistant cells (INS‐R) had lower expression of insulin signaling genes, including *Ir, Igf‐1r, Irs1*, *Irs2*, *Akt1*, *Akt2*, and *Gsk3b* (*p* < .05) (Figure 2). Densitometric analysis on WB at basal (unstimulated) and post‐insulin stimulation (100 nM, 30 min) in the Control showed a robust increase in Y1146‐pIRβ, Y458‐pPI3K p85, S473‐pAKT, and S9‐pGSK3B upon insulin stimulation (*p* < .05 vs. Control‐Basal) (Figure 3). Insulin resistance did not affect the basal phosphorylation of these proteins but markedly blunted their phosphorylation post‐insulin stimulation (*p* < .05 vs. Control‐Insulin stimulated), indicating defective signaling transduction.

**FIGURE 1:**
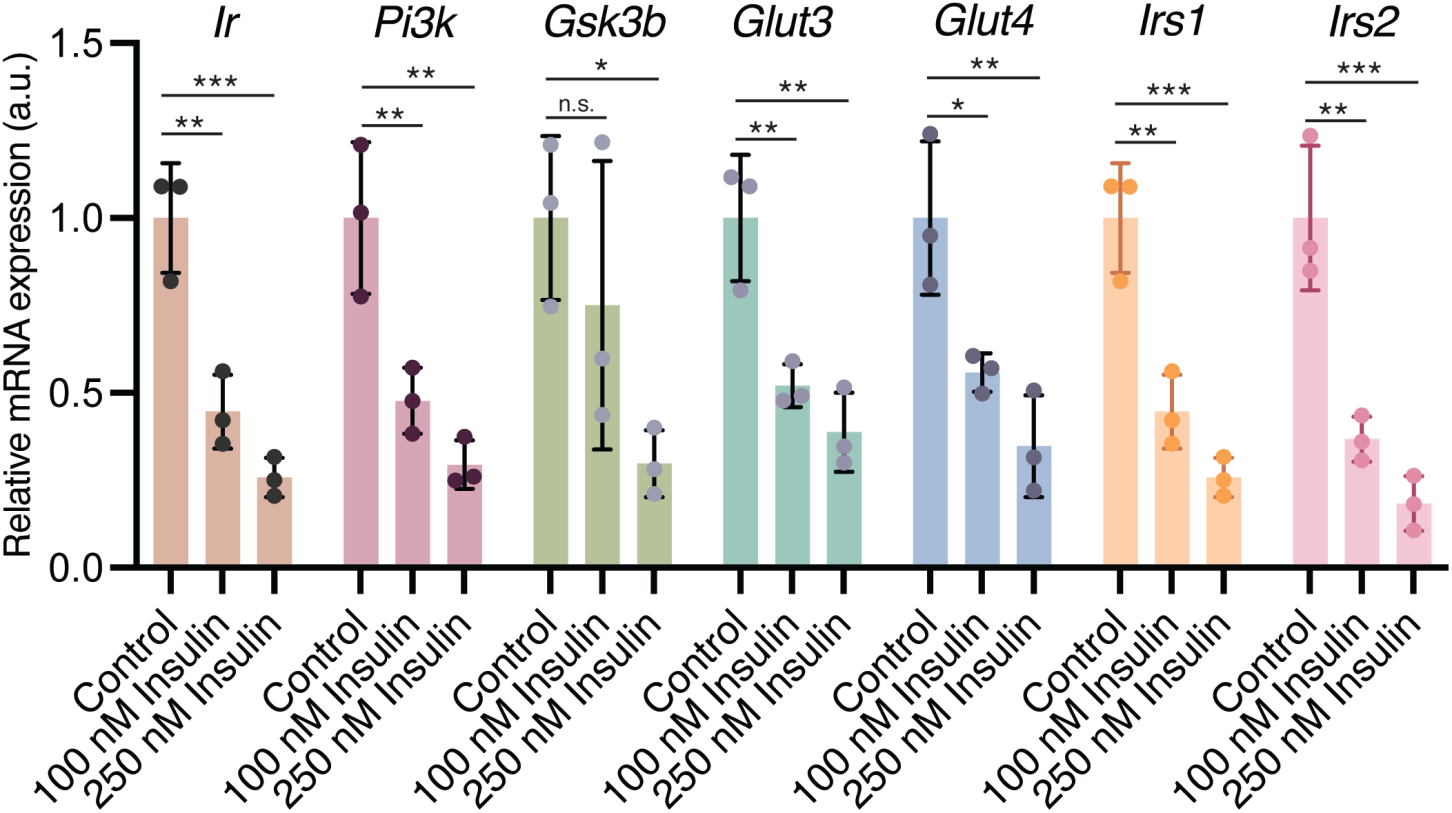
Subjecting differentiated SK‐N‐SH cells to high insulin media (100 and 250 nM) for 24 h downregulated mRNA expression of insulin signaling genes. Data are expressed as mean ± SD (*n* = 3), *p < .05, **p < .01 and ***p < .001, one‐way ANOVA with Dunnet's post‐hoc.

**FIGURE 2:**
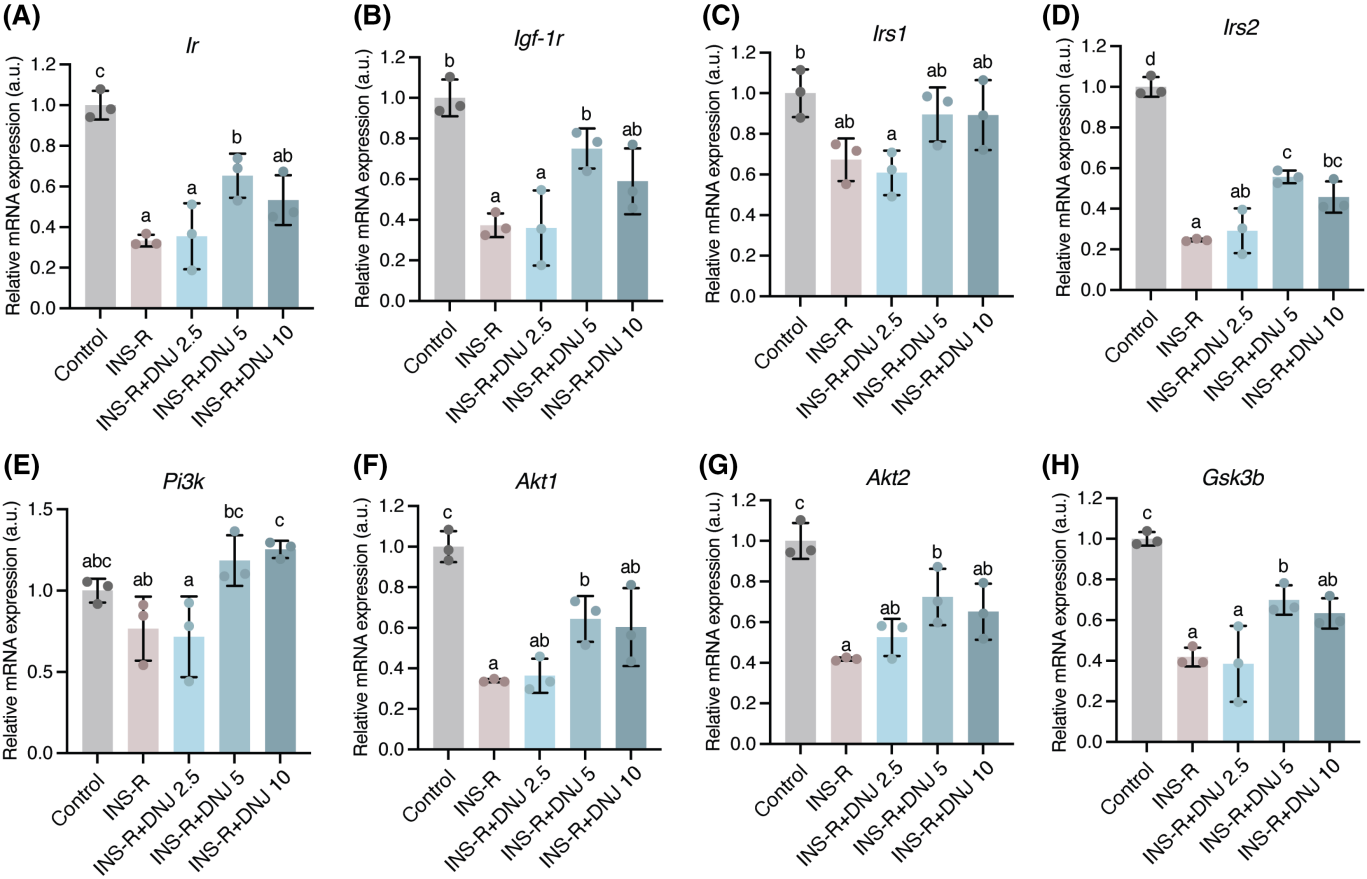
DNJ altered the expression of insulin signaling genes in insulin‐resistant SK‐N‐SH cells (INS‐R). Relative mRNA expression of (A) *Ir*, (B) *Igf‐1r*, (C) *Irs1*, (D) *Irs2*, (E) *Pi3k*, (F) *Akt1*, (G) *Akt2*, (H) *Gsk3b* with *Gapdh* as reference gene. Data are presented as mean ± SD (*n* = 3), ^a,b,c^ *p* < .05, one‐way ANOVA with Tukey's post‐hoc test.

**FIGURE 3:**
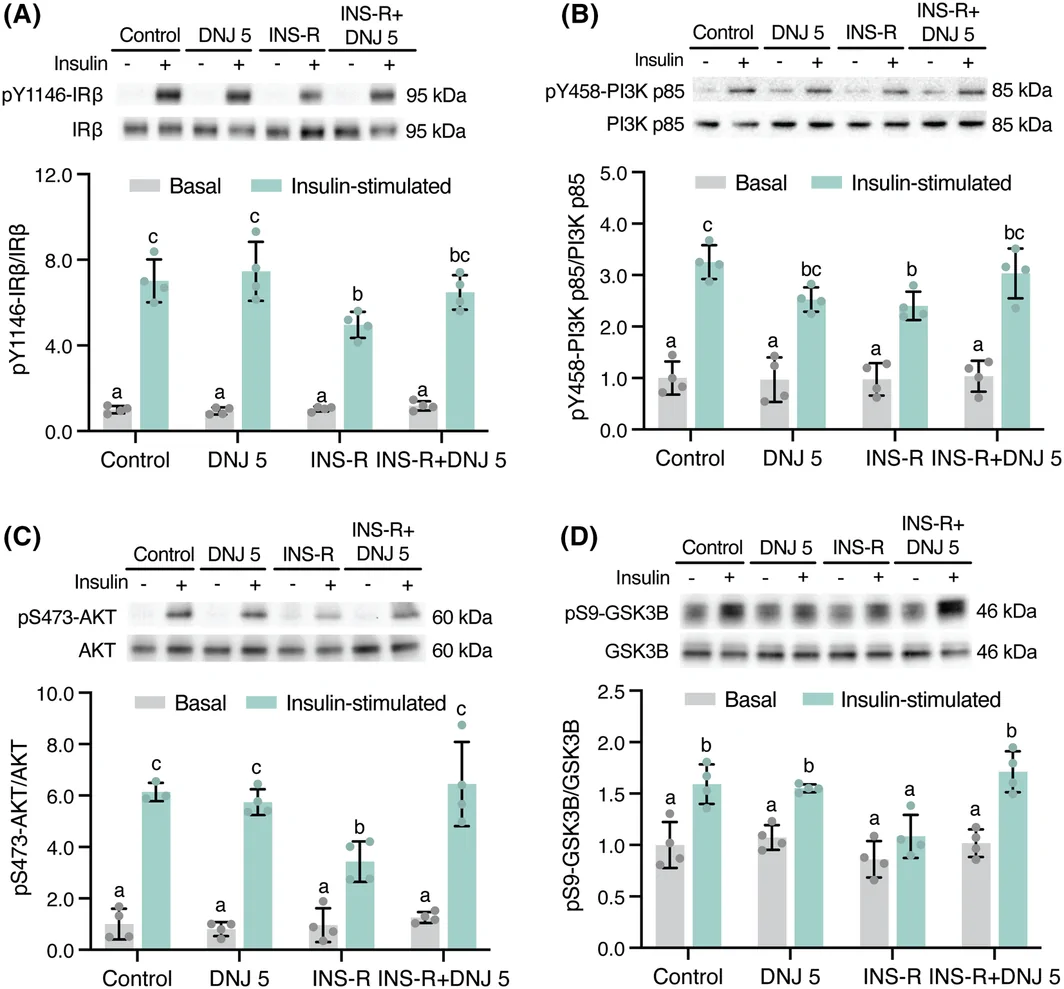
DNJ restored insulin‐induced phosphorylation of insulin signaling proteins in INS‐R cells. Representative western blot and densitometric analysis for (A) pY1146‐IRβ, (B) pY458‐PI3K p85, (C) pS473‐AKT, (D) pS9‐GSK3B at basal and post‐insulin stimulation. Data are presented as mean ± SD (*n* = 4), ^a,b,c^ *p* < .05, two‐way ANOVA with Tukey's post‐hoc test.

To assess the effect of DNJ on insulin signaling, we treated INS‐R cells with 2.5, 5, and 10 μM DNJ (INS‐R + DNJ 2.5, 5, and 10) for 24 h and measured the changes in the expression of insulin signaling genes. DNJ exhibited the most potent effect at 5 μM, where it increased the mRNA expression of *Ir*, *Igf‐1r*, *Irs2*, *Akt1*, *Akt2*, and *Gsk3b* (*p* < .05) and slightly elevated *Pi3k* (*p* = .062) in INS‐R. WB assay revealed higher post‐insulin expression of S9‐pGSK3B and S473‐pAKT (*p* < .05) in INS‐R cells treated with 5 μM DNJ (INS‐R + DNJ 5) compared to untreated INS‐R cells, with a slight rise in Y1146‐pIRβ and Y458‐pPI3K p85 (*p* = .093 and .209), while their basal levels remained similar. DNJ did not induce any discernible changes in insulin‐sensitive cells (DNJ 5), except for a slight decrease in post‐insulin level of Y458‐pPI3K p85 (*p* = .101 vs. Control). Our data argue that DNJ could reverse neuronal insulin resistance by altering the expression and phosphorylation status of key insulin signaling molecules without affecting insulin‐sensitive cells.

### 3.2 DNJ promoted 2‐DG uptake and upregulated mRNA expression of glucose transporters in insulin‐resistant SK‐N‐SH

Insulin signaling is crucial for the proper regulation of glucose uptake. Defects in insulin signaling impair the membrane trafficking of insulin‐responsive glucose transporters such as GLUT4 and disrupt glucose entry. Here, we studied the effect of insulin resistance on the mRNA expression of glucose transporters (GLUT) and cellular 2‐DG uptake. Compared to the Control, INS‐R had reduced *Glut3* and *Glut4* expression, lower basal and post‐insulin 2‐DG uptake, and an overall lower fold‐change in 2‐DG uptake (*p* < .05) (Figure 4), indicating impaired glucose uptake machinery. Treating INS‐R cells with 5 μM DNJ (INS‐R + DNJ 5) increased *Glut3* and *Glut4* expression, elevated post‐insulin 2‐DG level, and increased the fold‐change in insulin‐induced 2‐DG uptake (*p* < .05 vs. INS‐R). These data altogether suggest that DNJ ameliorates the defects in glucose uptake machinery by reversing insulin resistance and regulating the expression of glucose transporters.

**FIGURE 4:**
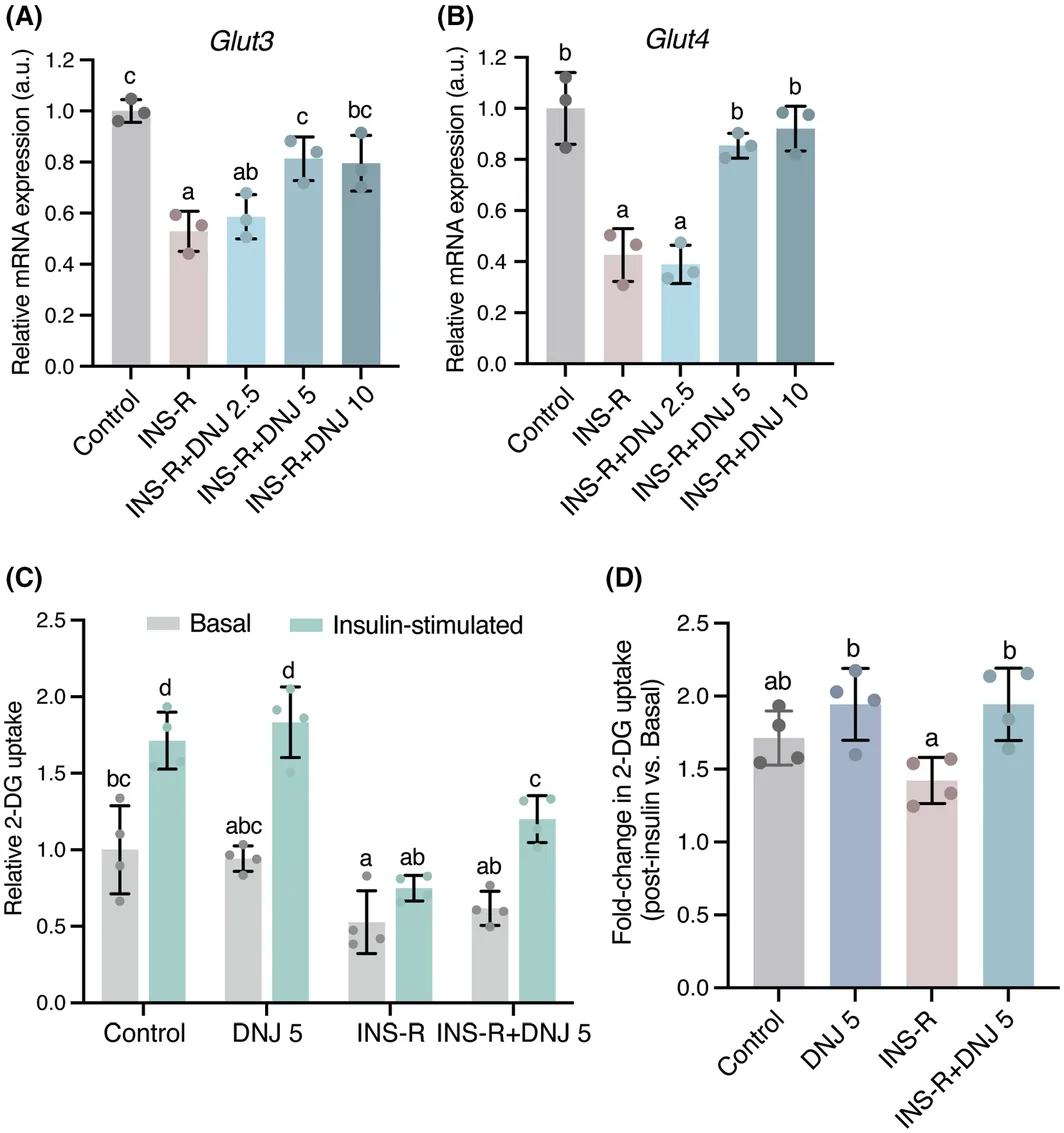
DNJ increased insulin‐induced glucose uptake in INS‐R cells. mRNA level of (A) *Glut3* and (B) *Glut4* relative to *Gapdh*. (C) Relative 2‐DG uptake at basal and post‐insulin stimulation. (D) Fold‐change in 2‐DG uptake (post‐insulin /basal). Data are presented as mean ± SD (*n* = 3–4), ^a,b,c,d^ *p* < .05, one‐way ANOVA (A, B, and D), or two‐way ANOVA (C) with Tukey's post‐hoc test.

### 3.3 DNJ mitigated AD‐like pathologies driven by insulin resistance in SK‐N‐SH neuroblastoma

Increasing studies have proposed insulin resistance as a driver for aberrant tau phosphorylation and Aβ pathologies in AD. Here, we estimated the expression of S202‐pTAU in cell lysates using WB and measured the Aβ_1‐42_ level in cell‐conditioned media using ELISA. To identify the mechanisms underlying Aβ accumulation in INS‐R cells, we measured the expression of amyloid precursor protein (APP), whose cleavage forms Aβ peptide, and the mRNA expression of insulin‐degrading enzyme (*Ide*) that degrades insulin and Aβ. Our data shows that INS‐R had a higher Aβ_1‐42_ level and lower mRNA expression of *Ide* than Control (*p* < .05) (Figure 5), with relatively similar APP expression. The post‐insulin expression of S202‐pTAU was also higher in INS‐R (*p* < .05 vs. Control). Treating INS‐R cells with 5 μM DNJ (INS‐R + DNJ 5) resulted in lower post‐insulin expression of S202‐pTAU (*p* < .05), reduced Aβ_1‐42_ level (*p* = .051), and higher *Ide* expression (*p* < .05) compared to untreated INS‐R cells. Treating insulin‐sensitive cells with DNJ did not affect Aβ_1‐42_ levels and S202‐pTAU expression.

**FIGURE 5:**
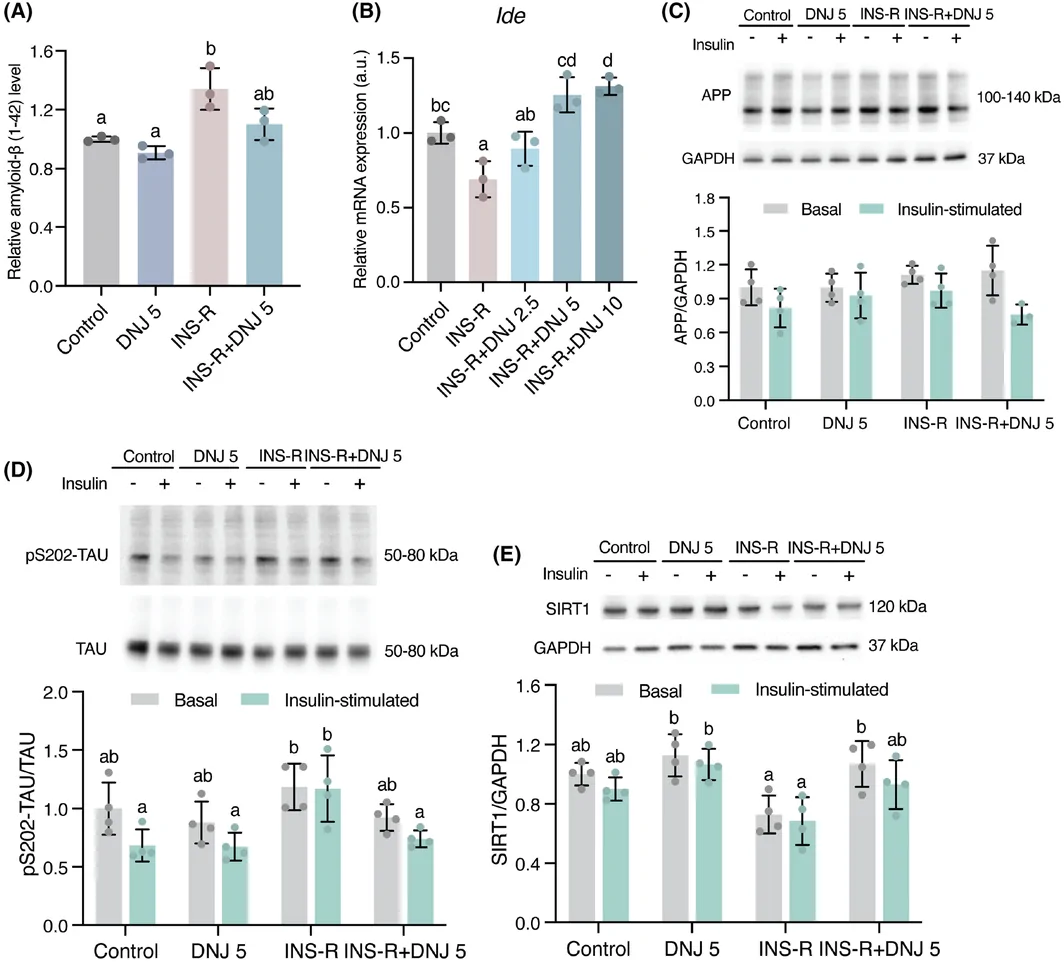
DNJ reduced tau phosphorylation and amyloid‐β (Aβ) levels in INS‐R cells. (A) Aβ (1–42) level in cell‐conditioned media. (B) mRNA expression of *Ide* relative to *Gapdh*. (C) Representative western blots and densitometric analysis for APP; (D) pS202‐TAU/TAU ratio; and (E) SIRT1 at basal and post‐insulin stimulation. Data are presented as mean ± SD (*n* = 3–4), ^a,b,c,d^ *p* < .05, one‐way ANOVA (A and B) or two‐way ANOVA (C–E) with Tukey's post‐hoc test.

In addition to the classical AD markers, we estimated the expression of SIRT1, a deacetylase involved in metabolic regulation, stress response, and longevity in various tissues, including the brain.^32^ Our data shows a slight reduction in SIRT1 expression in INS‐R (*p* = .105 and .315 vs. Control for basal and post‐insulin). Five micromolar DNJ treatments increased SIRT1 expression in INS‐R (*p* < .05 and .184 at basal and post‐insulin) but had no impact on insulin‐sensitive cells (DNJ 5). These data suggest that DNJ confers neuroprotection in our cell model by reducing tau phosphorylation, promoting Aβ_1‐42_ clearance, and increasing SIRT1 expression.

### 3.4 DNJ uptake by SK‐N‐SH cells

Like most bioactive compounds, the ability of DNJ to demonstrate its functional properties relies on its cellular uptake. Hence, we measured cellular uptake of DNJ over time within 24 h of incubation in the media containing 5 μM DNJ (the optimal level for reversing insulin resistance). Cells were harvested at different time points (6, 12, 18, 24 h), and DNJ was isolated from cell lysates for detection and quantification using the HPLC‐MS/MS system. MRM chromatograms from MS/MS detection showed a gradual increase in intracellular DNJ levels within 18 h before hitting a plateau at 24 h (Figure 6), thereby confirming the ability of DNJ to be taken up by SK‐N‐SH cells.

**FIGURE 6:**
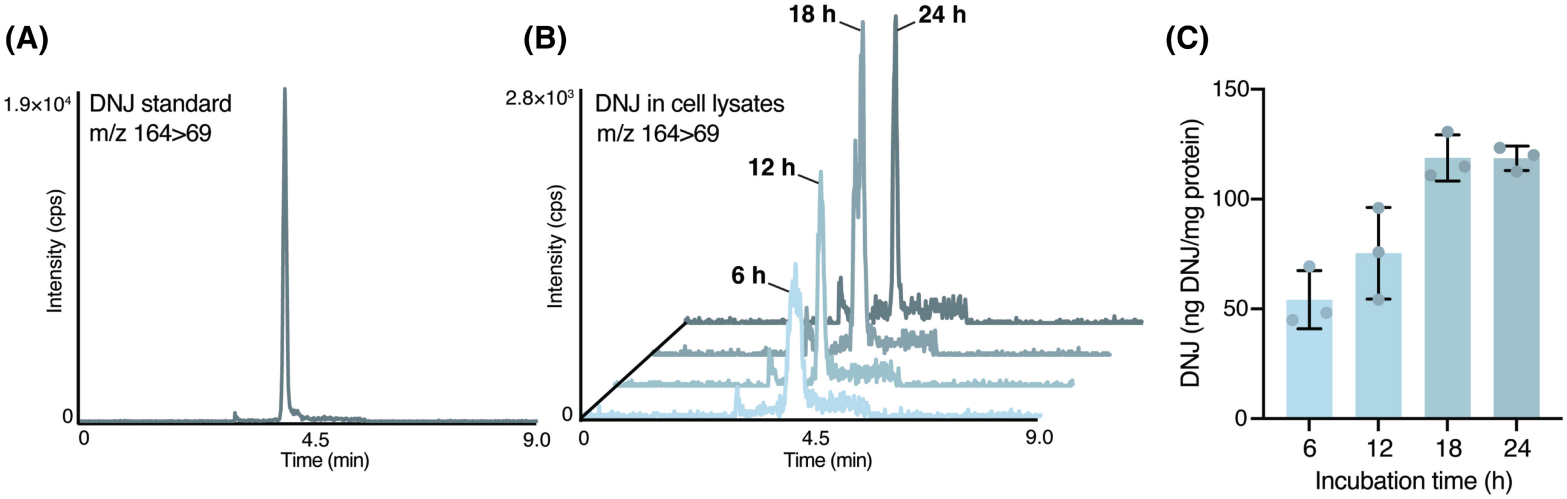
DNJ uptake by SK‐N‐SH cells. The HPLC‐MS/MS chromatogram of (A) DNJ standard (50 ng/mL) and (B) DNJ in cell extract after incubation in media containing DNJ (5 μM) for 6, 12, 18, and 24 h. (C) The quantity of DNJ detected in cell extract over time. Data are presented as mean ± SD (*n* = 3).

## 4 DISCUSSION

Brain insulin resistance has emerged as one of the risk factors for AD. Changes associated with insulin resistance have been detected in the post‐mortem brain of AD, ranging from aberrant expression and distribution of IR, IGF‐1R, and IRS1/2 in the temporal cortex^5^ to reduced responses of pY1150/1151‐IRβ, pS473‐AKT1, and pS9‐GSK3B to insulin stimuli in hippocampus.^4^ Impaired central insulin signaling often coincides with—and even precedes—the cognitive deficits and classic rise in Aβ plaques and tau deposits,^4^, ^33^ thus opening new avenues of treatment by repurposing peripheral insulin sensitizers. Here, we assessed the benefits of DNJ in resolving insulin resistance and associated AD pathologies in insulin‐resistant SK‐N‐SH neuroblastoma, a representative model of human neuronal insulin resistance. We used DNJ levels ranging from 2.5–10 μM as they are equivalent to plasma DNJ levels in humans after oral intake of DNJ at effective doses.^12^, ^34^ DNJ uptake by SK‐N‐SH cells peaked within 18 h of incubation in media with 5 μM DNJ, thus confirming the availability of DNJ to perform its functions in our cell model (Figure 7).

**FIGURE 7:**
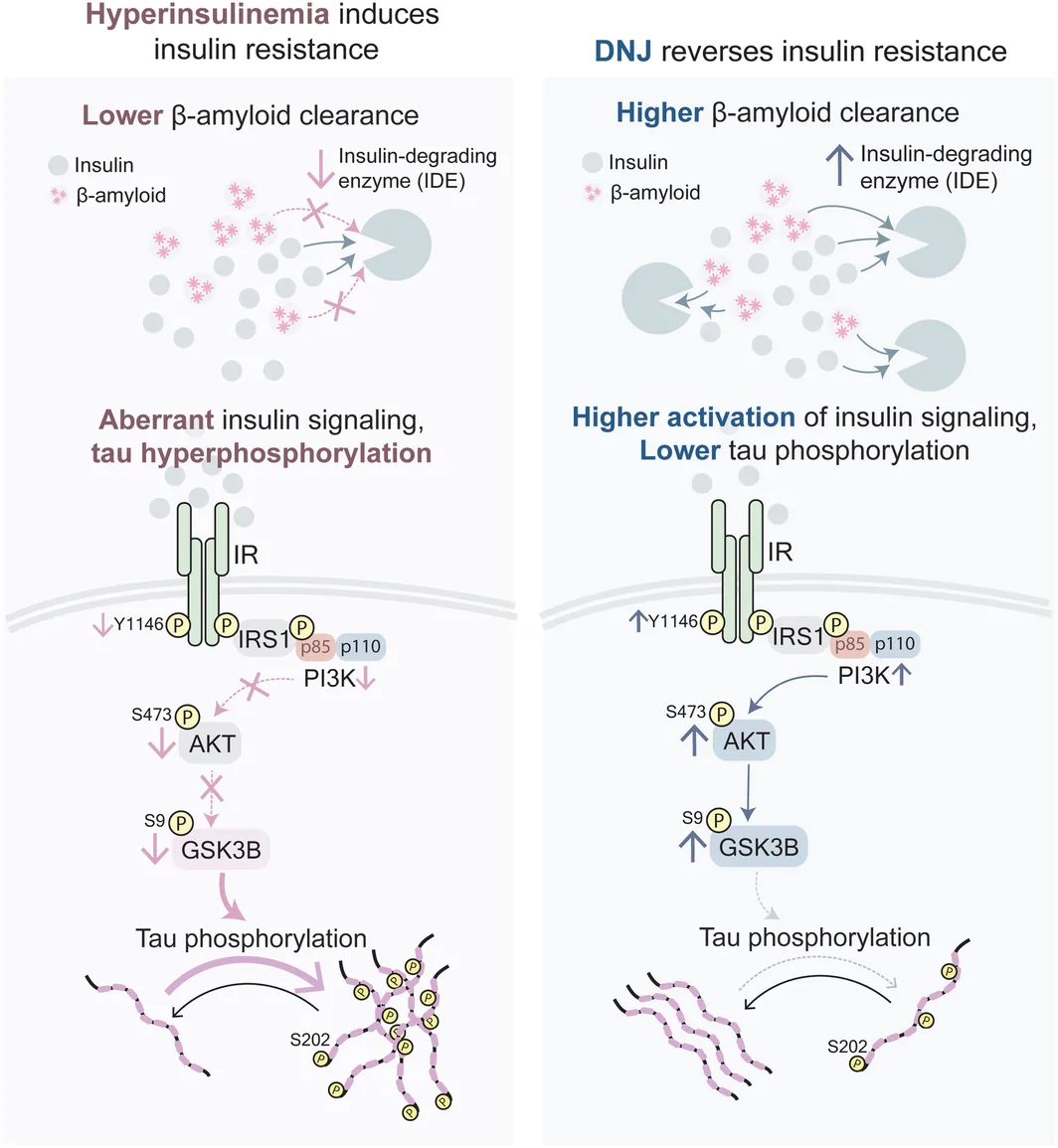
Schematic diagram predicting DNJ's impact on insulin signaling and AD markers in insulin‐resistant SK‐N‐SH. DNJ enhances insulin‐induced Y1146 phosphorylation of IRβ, triggering phosphorylation of downstream Y458‐PI3K p85, S473‐AKT, and S9‐GSK3B. S9 phosphorylation of GSK3B inhibits its kinase activity towards tau and reduces the rate of tau phosphorylation. DNJ also lowers Aβ_1‐42_ by increasing the expression of IDE, an enzyme responsible for degrading insulin and Aβ.

Our insulin‐resistant model (INS‐R) exhibited impaired insulin‐regulated IR/PI3K/AKT signaling transduction, marked by reduced expression of key insulin signaling genes (*Ir*, *Igf‐1r*, *Irs2*, *Akt1*, *Akt2*, *Gsk3b*) and impaired phosphorylation of IR and its downstream molecules (PI3K, AKT, and GSK3B). DNJ effectively reversed insulin resistance by increasing the expression of insulin signaling genes and promoting insulin‐induced phosphorylation of IR, PI3K, AKT, and GSK3B. While the insulin‐sensitizing effect of DNJ on neurons or the brain has yet to be described, its impact on the periphery has been well‐documented. Notably, our study in diet‐induced obese mice demonstrated higher expression of insulin signaling genes (*Irs1*, *Akt2*, *Pten*) in white adipose tissue after oral DNJ intake (10 mg/kg BW/day) for 10 weeks.^18^ Another study by Liu et al.^19^ reported higher phosphorylation levels of proteins in the PI3K/AKT signaling and a rise in membrane GLUT4 levels in the skeletal muscle of db/db mice after 4 weeks of DNJ intake (40 or 80 mg DNJ/kg BW/day). Using a similar model, one study reported higher expression of S473‐AKT and S9‐GSK3B in the liver preceding the decrease in GSK3B activity.^20^ Collectively, our data substantiate the localized impact of DNJ on the IR/PI3K/AKT signaling and that this mechanism is relevant in our cell model of neuronal insulin resistance.

Like the periphery, insulin signaling orchestrates various physiological functions in the CNS. Most notably, it promotes glucose uptake by redistributing GLUT4 to the plasma membrane (PM).^35^, ^36^ Beyond its impact on insulin signaling, prolonged hyperinsulinemia reduced basal and insulin‐stimulated glucose uptake in our cell model and downregulated the mRNA expression of *Glut3* (predominant GLUT isoform in brain^37^) and *Glut4* (preferentially expressed in the cerebellum and hippocampus^35^, ^36^). Unlike GLUT4, activation of insulin signaling has little influence on GLUT3 distribution to PM.^38^ Thus, it is likely that defective insulin signaling and lower *Glut4* in INS‐R were responsible for reduced insulin‐regulated glucose uptake, while downregulation of *Glut3* might have a more pronounced effect at basal. Past studies have reported tissue‐specific action of DNJ on GLUT4 biology, with studies on 3 T3‐L1 adipocytes showing higher GLUT4 expression after treatment with DNJ at 5 μM^39^ or as low as 0.5 μM.^40^ This is in line with an in vivo report in db/db mice wherein intravenous DNJ administration (40 and 80 mg/kg BW/day) increased membrane GLUT4 expression in skeletal muscle after four weeks without changing total GLUT4 expression.^19^ Our data aligns with the evidence from the periphery, as DNJ increases both *Glut3* and *Glut4* mRNA in our neuronal insulin‐resistant model. Despite its modest impact on the basal 2‐DG uptake in insulin‐resistant cells, DNJ induced greater 2‐DG uptake upon insulin stimuli, with a response magnitude comparable to the Control. Our data thereby argues that DNJ improves insulin‐regulated glucose uptake by restoring insulin sensitivity and upregulating the expression of insulin‐responsive *Glut4* mRNA.

Brain insulin resistance has been reported as one of the drivers for AD pathologies, including aberrant tau phosphorylation and Aβ accumulation.^2^ Hyperinsulinemia induced higher tau phosphorylation in SK‐N‐SH neuroblastoma, consistent with the framework in which insulin resistance lowers AKT‐induced GSK3B phosphorylation at S9, which in turn enhances GSK3B kinase activity towards tau.^41^ These processes underpin the subsequent changes in our study, where DNJ increased pS9‐GSK3B (indicative of lower GSK3B activity) in INS‐R cells and reduced phosphorylated tau. Hyperinsulinemia also increased the Aβ_1‐42_ level in cell‐conditioned media and reduced the intracellular expression of *Ide*, an enzyme implicated in insulin and Aβ degradation. Insulin resistance supposedly reduces the expression and catalytic activity of IDE^10^ or lowers its affinity for Aβ due to competition with insulin.^9^ This ultimately interferes with Aβ clearance and might account for the higher Aβ_1‐42_ level observed in our insulin resistance model. Interestingly, DNJ not only reduced Aβ_1‐42_ level in the cell media of insulin‐resistant cells but also upregulated *Ide* expression. Prior data from SAMP8 mice demonstrated that repeated intake of DNJ for 2 months at a high dose (160 mg/kg BW/day) reduces hippocampal Aβ deposition by alleviating neuroinflammation and increasing the expression of BACE1, an APP‐cleaving enzyme involved in Aβ anabolism.^25^ While we do not have sufficient data to determine the impact of DNJ on Aβ anabolism, our study introduces a novel perspective whereby DNJ may promote Aβ clearance by regulating the expression of *Ide*.

Aside from the classical AD markers, we assessed the changes in the expression of SIRT1 expression due to the increasing interest in its role in various neurological processes.^32^ To support this notion, findings from post‐mortem brain, plasma, and lymphoblastoid cell lines have revealed a progressive decline in SIRT1 parallel to the severity of AD,^42^, ^43^ with studies suggesting that SIRT1 promotes tau deacetylation and hinders propagation of tau aggregates.^44^ Our data shows that hyperinsulinemia slightly reduces SIRT1 expression; such a phenomenon has been observed in some models of insulin resistance, though the causal relationship between the two is not well‐elucidated.^45^ At least two studies report the effect of DNJ on SIRT1: E et al.^46^ reported higher SIRT1 expression in mice colorectal cancer tissues after 14 weeks of DNJ intake, while our previous study showed higher SIRT1 expression in the white adipose tissue of obese mice after 10 weeks of DNJ intake.^18^ In line with these findings, our data shows a slight increase in SIRT1 expression in INS‐R after DNJ treatment, which may partially explain the reduction in tau phosphorylation. These data overall provide additional mechanistic layer to our study, whereby the insulin‐sensitizing effect of DNJ is integral for mitigating AD pathologies.

## 5 CONCLUSION

Our findings highlight the ability of DNJ to resolve neuronal insulin resistance and counter the associated rise in AD markers in SK‐N‐SH neuroblastoma. At a physiologically relevant level (5 μM), DNJ increased glucose uptake by promoting insulin‐regulated IR/PI3K/AKT signaling and increasing GLUT mRNA in our insulin resistance model. Notably, DNJ lowered tau phosphorylation, presumably by curbing GSK3B tau kinase activity via increasing AKT‐mediated S9 phosphorylation of GSK3B. DNJ also increased the mRNA expression of *Ide*—an enzyme crucial to Aβ degradation—resulting in lower Aβ levels in cell‐conditioned media. Considering the evidence from our study and prior investigations employing a more severe aging model,^24^, ^25^ we propose using DNJ for preventive measures rather than treatment for late‐stage AD. This not only holds better potential for delaying AD but also requires a lower DNJ dosage. One limitation of our study is the incomplete elucidation of the intricate mechanisms underpinning DNJ's neuroprotective efficacy (e.g., DNJ's impact on IDE catalytic activity and interaction with insulin signaling molecules). Thus, future studies should delve deeper into these topics and, more importantly, validate the findings using suitable in vivo models. Altogether, our study provides a mechanistic understanding of the neuroprotective benefits of DNJ and presents it as a viable therapeutic choice for preserving brain insulin sensitivity and delaying the onset of AD.

## AUTHOR CONTRIBUTIONS

*Isabella S. Parida*: Conceptualization, Methodology, Formal Analysis, Investigation, Validation, Data Curation, Resources. *Soo Takasu*, *Junya Ito, Takahiro Eitsuka*: Methodology, Data Curation, Validation. *Kiyotaka Nakagawa*: Supervision, Validation, Resources. All authors were involved in drafting and revising the manuscript.

## DISCLOSURES

The authors declare no conflicts of interest.

## Supporting information


Figure S1.

Figure S2.

Figure S3.

Table S1.

Table S2.

## Data Availability

The data that supports the findings of this study is available from the corresponding author upon reasonable request.
