# Supplementary material for: 1‐Deoxynojirimycin attenuates pathological markers of Alzheimer's disease in the in vitro model of neuronal insulin resistance
Source: FASEB J. 2024 Jul 9;38(13):e23800. doi: 10.1096/fj.202302600R (PMC13687917; doi:10.1096/fj.202302600R)
Supplement: Supplementary file 1 [file FSB2-38-e23800-s001.pdf]

Supplementary Table 2. List of primary and secondary antibodies used in Western Blotting

Antibody name	Reactivity	CAT number	Dilution
Insulin Receptor β	Rabbit	23413	1:2000
Phospho-Insulin Receptor β (Tyr1146)	Rabbit	3021	1:1000
IRS-1	Rabbit	2390	1:2000
Phospho-IRS-1 (Ser307)	Rabbit	2381	1:2000
PTEN	Rabbit	9188	1:2000
PI3K p85	Rabbit	4257	1:1000
Phospho-PI3 Kinase p85 (Tyr458)	Rabbit	17366	1:1000
Akt (pan)	Rabbit	4691	1:2000
Phospho-Akt (Ser473)	Rabbit	4060	1:2000
GSK-3β	Rabbit	12456	1:2000
Phospho-GSK-3β (Ser9)	Rabbit	5558	1:2000
Tau	Rabbit	46687	1:2000
Phospho-Tau (Ser202)	Rabbit	39357	1:1000
Anti-rabbit IgG, HRP-linked Antibody	Rabbit	7074	1:10,000
