# Supplementary material for: 1‐Deoxynojirimycin attenuates pathological markers of Alzheimer's disease in the in vitro model of neuronal insulin resistance
Source: FASEB J. 2024 Jul 9;38(13):e23800. doi: 10.1096/fj.202302600R (PMC13687917; doi:10.1096/fj.202302600R)
Supplement: Supplementary file 2 [file FSB2-38-e23800-s002.pdf]

Supplementary Table 1. List of primers used in RT-qPCR

Gene name	Genbank accession number	Forward primer (5'-3')	Reverse primer (5'-3')
NeuroD (*NeuD*)	NM_002500.5	CCGTCCGCCGAGTTTG	GCGGTGCCTGAGAAGATTG
Synaptophysin (*Syp*)	NM_003179.3	ATTGTGCCAACAAGACCGAGAGT	CAGGAAGATGTAGGTGGCCAGAG
Microtubule associated protein 2 (*Map2*)	NM_001375529.1	CATGGGTCACAGGGCACCTATTC	GGTGGAGAAGGAGGCAGATTAGCTG
Insulin receptor (*Ir*)	NM_000208.4	CAACATTCGAGGAGGCAACAATC	CTCGAATCAGACGTAACTTCCGG
Insulin-like growth factor-1 receptor (*Igf-1r*)	NM_000875.5	ACTTACTCGGACGTCTGGTCCTTC	ATCTTGGGGTTATACTGCCAGCAC
Insulin receptor substrate 1 (*Irs1*)	NM_005544.3	TGCTGGGGGTTTGGAGAATG	GGCACTGTTTGAAGTCCTTGACC
Insulin receptor substrate 2 (*Irs2*)	NM_003749.3	AAAATTGGCGGAGCAAGGC	ATGTTCAGGCAGCAGTCGAGAG
Phosphoinositide 3-kinase (*Pi3k*)	NM_006218.4	TGGATGCTCTACAGGGCTTT	GTCTGGGTTCTCCCAATTCA
Phosphatase and tensin homolog (*Pten*)	NM_000314.8	TTTGAAGACCATAACCCACCAC	ATTACACCAGTTCGTCCCTTTC
AKT serine/threonine kinase 1 (*Akt1*)	NM_001382431.1	TCTATGGCGCTGAGATTGTG	CTTAATGTGCCCGTCCTTGT
AKT serine/threonine kinase 2 (*Akt2*)	NM_001243027.3	TATACCGCGACATCAAGCTG	GGTCCCACAGAAGGTTTTCA
Glycogen synthase kinase-3b (*Gsk3b*)	NM_002093.4	CTAACACCACTGGAAGCTTGTGC	GATGGTAGCCAGAGGTGGATTAC
Insulin-degrading enzyme (*Ide*)	NM_004969.3	ATGTTCTTGCCAGGGAAATG	CAGAGTTTTGCAGCCATGAA
Glucose transporter 3 (*Glut3*)	NM_006931.3	TCCCCTCCGCTGCTCACTATTT	ATCTCCATGACGCCGTCCTTTC
Glucose transporter 4 (*Glut4*)	NM_001042.3	CCCTCAGAAGGTGATTGAACAG	AGAGATGATACCAATGAGGAAGG
Glyceraldehyde-3-phosphate dehydrogenase (*Gapdh*)	NM_001357943.2	TCGACAGTCAGCCGCATCTTCTTT	ACCAAATCCGTTGACTCCGACCTT
