# Supplementary material for: 1‐Deoxynojirimycin attenuates pathological markers of Alzheimer's disease in the in vitro model of neuronal insulin resistance
Source: FASEB J. 2024 Jul 9;38(13):e23800. doi: 10.1096/fj.202302600R (PMC13687917; doi:10.1096/fj.202302600R)

Supplementary Fig. 2. Cell viability of SK-N-SH as affected by varying concentrations of **A)** DNJ (2.5-1000 µM) and **B)** insulin (50-250 nM). Data are expressed as mean ± SD (n = 6), $^{*}P<0.05$, one-way ANOVA with Dunnet's post-hoc.

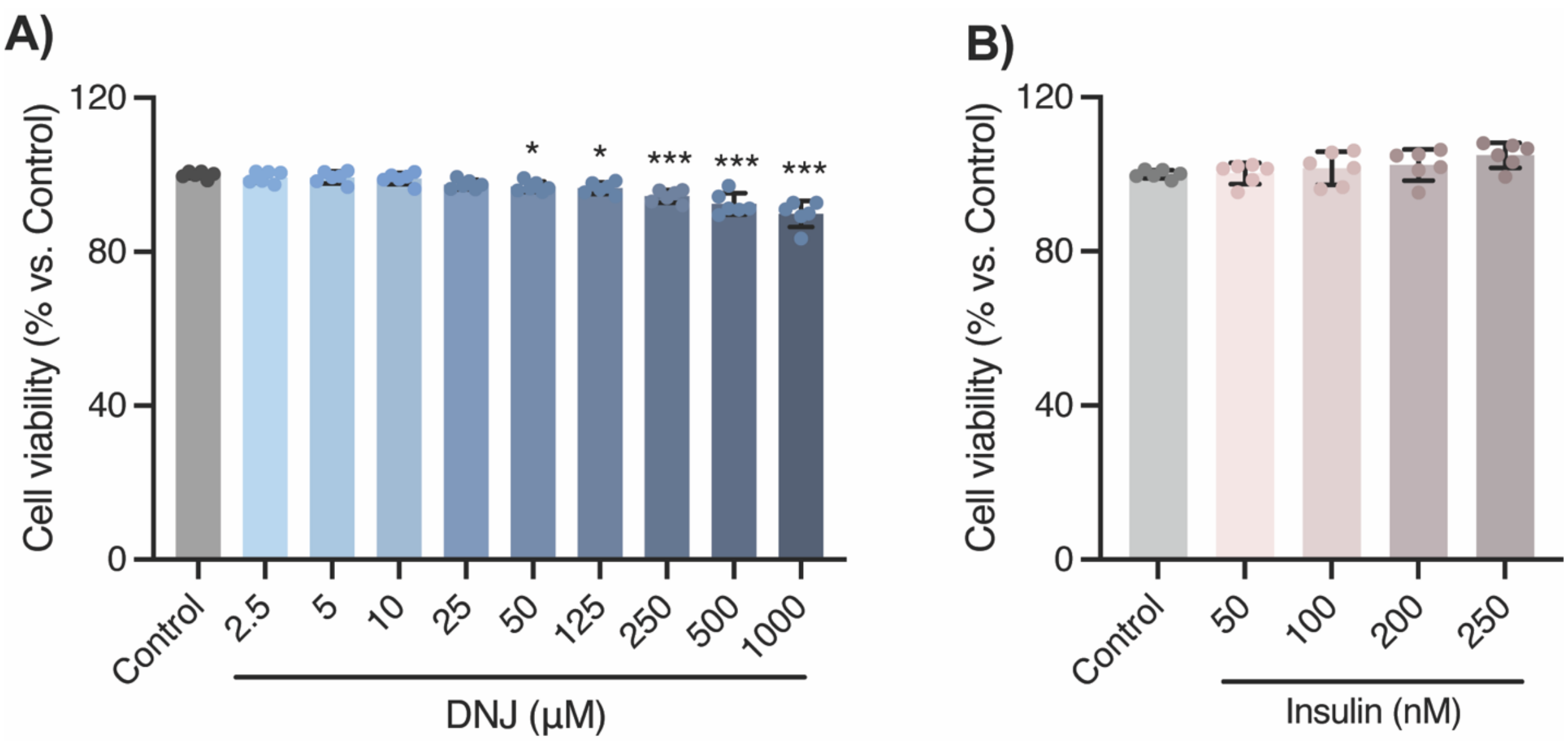

Supplement: Supplementary file 3 [file FSB2-38-e23800-s003.pdf]
