# Supplementary material for: 1‐Deoxynojirimycin attenuates pathological markers of Alzheimer's disease in the in vitro model of neuronal insulin resistance
Source: FASEB J. 2024 Jul 9;38(13):e23800. doi: 10.1096/fj.202302600R (PMC13687917; doi:10.1096/fj.202302600R)

Supplementary Fig. 1. Retinoic acid (10 μM) and gradual serum deprivation induced differentiation of SK-N-SH cells to neuronal-like phenotypes. Relative mRNA expression of *NeuD*, *Syp*, and *Map2* as differentiation markers. Data are presented as mean ± SD (n=3), *P<0.05, **P<0.01 (Student's t-test).

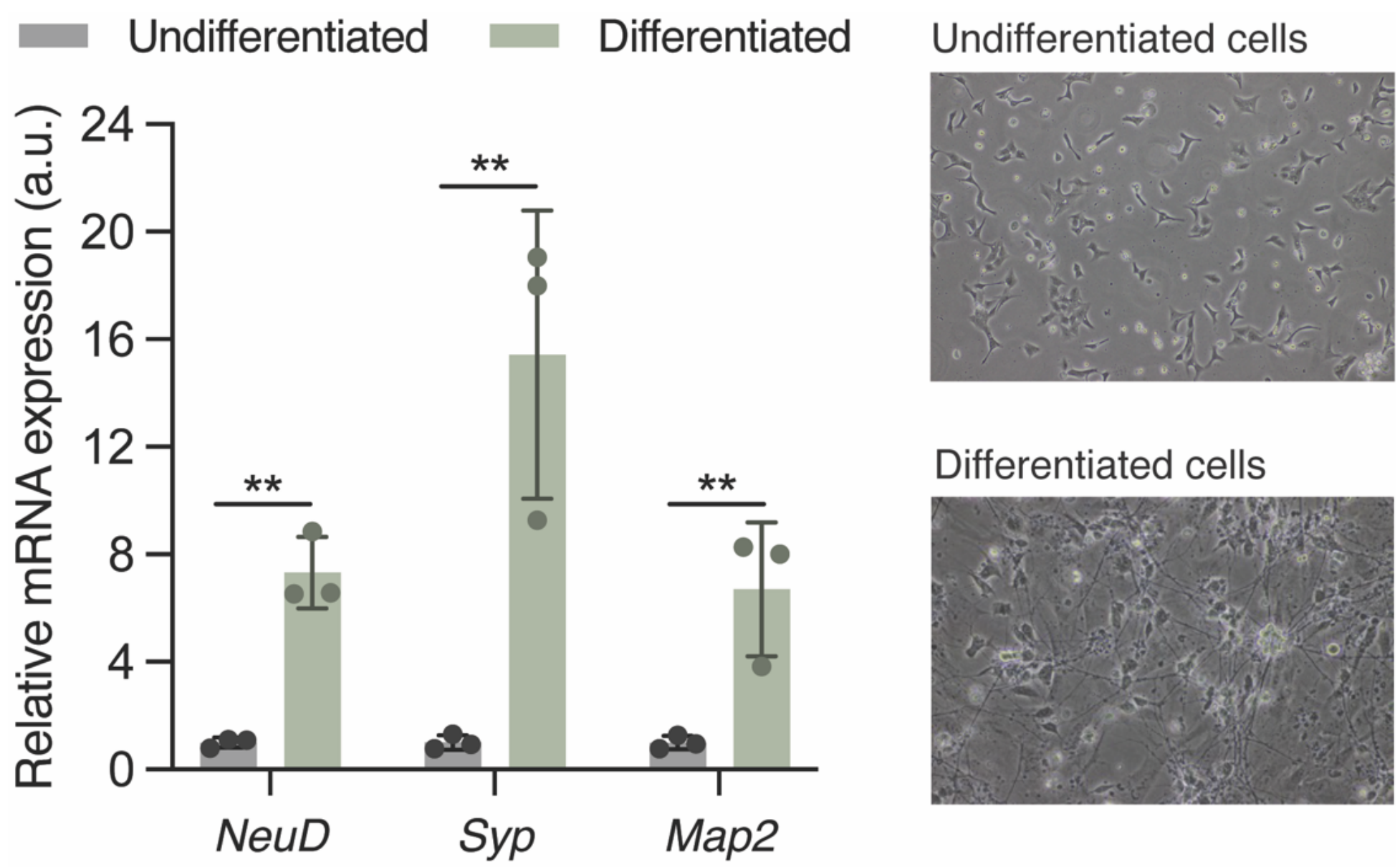

Supplement: Supplementary file 4 [file FSB2-38-e23800-s004.pdf]
