# Supplementary material for: 1‐Deoxynojirimycin attenuates pathological markers of Alzheimer's disease in the in vitro model of neuronal insulin resistance
Source: FASEB J. 2024 Jul 9;38(13):e23800. doi: 10.1096/fj.202302600R (PMC13687917; doi:10.1096/fj.202302600R)

Supplementary Fig. 3. Western blots for basal and insulin-stimulated expression of pY1146-IRβ pY458-PI3K p85, pS473-AKT, pS9-GSK3B, pS202-TAU, APP, SIRT1 for all replicates.

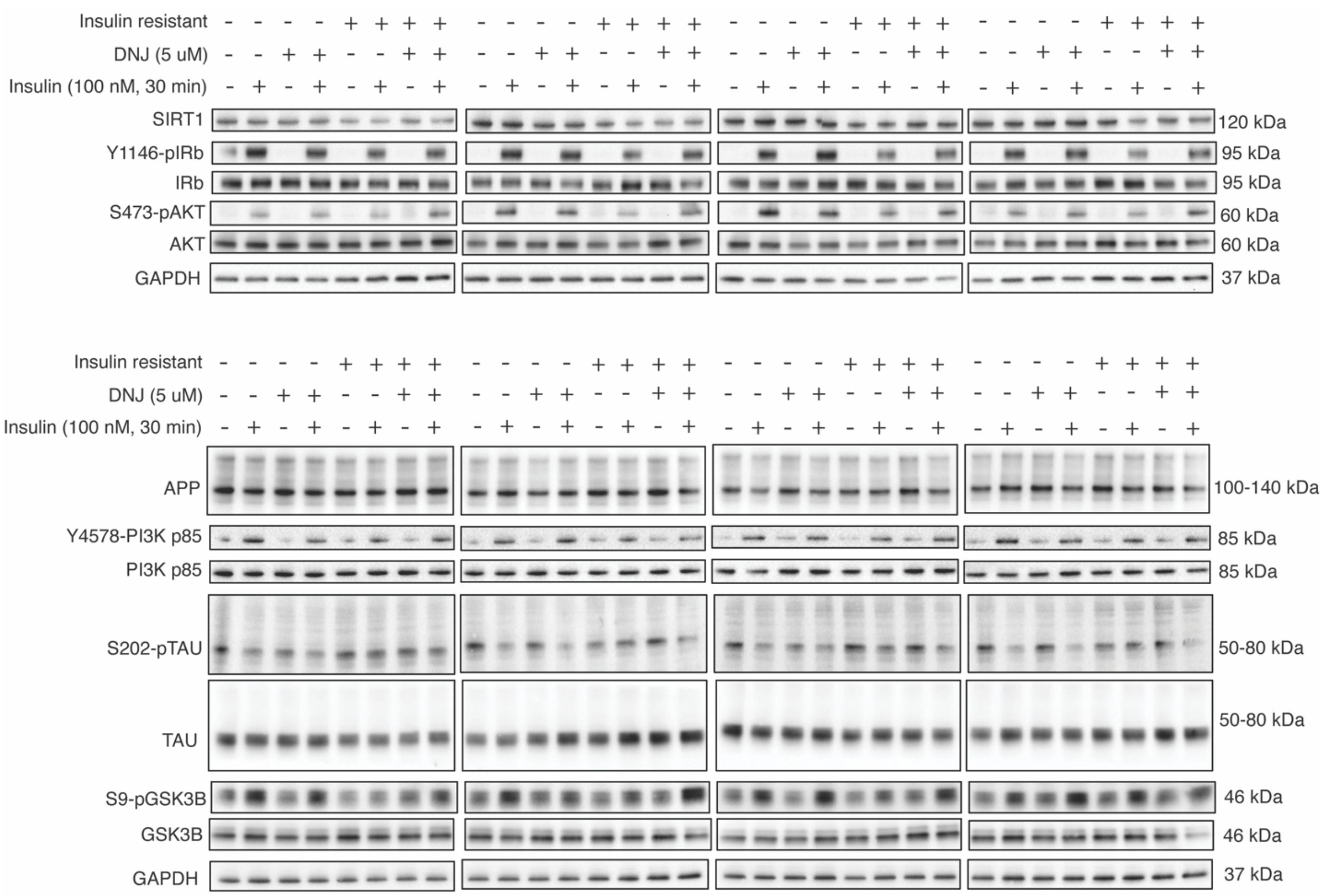

Supplement: Supplementary file 5 [file FSB2-38-e23800-s005.pdf]
